# Low Dimensional Discriminative Representation of Fully Connected Layer Features Using Extended LargeVis Method for High-Resolution Remote Sensing Image Retrieval

**DOI:** 10.3390/s20174718

**Published:** 2020-08-21

**Authors:** Zheng Zhuo, Zhong Zhou

**Affiliations:** State Key Laboratory of Virtual Reality Technology and Systems, School of Computer Science and Engineering, Beihang University, Beijing 100191, China; zzhuo@buaa.edu.cn

**Keywords:** high-resolution remote sensing image retrieval, extended LargeVis, ResNet50, channel attention mechanism, fully connected layer features

## Abstract

Recently, there have been rapid advances in high-resolution remote sensing image retrieval, which plays an important role in remote sensing data management and utilization. For content-based remote sensing image retrieval, low-dimensional, representative and discriminative features are essential to ensure good retrieval accuracy and speed. Dimensionality reduction is one of the important solutions to improve the quality of features in image retrieval, in which LargeVis is an effective algorithm specifically designed for Big Data visualization. Here, an extended LargeVis (E-LargeVis) dimensionality reduction method for high-resolution remote sensing image retrieval is proposed. This can realize the dimensionality reduction of single high-dimensional data by modeling the implicit mapping relationship between LargeVis high-dimensional data and low-dimensional data with support vector regression. An effective high-resolution remote sensing image retrieval method is proposed to obtain stronger representative and discriminative deep features. First, the fully connected layer features are extracted using a channel attention-based ResNet50 as a backbone network. Then, E-LargeVis is used to reduce the dimensionality of the fully connected features to obtain a low-dimensional discriminative representation. Finally, L2 distance is computed for similarity measurement to realize the retrieval of high-resolution remote sensing images. The experimental results on four high-resolution remote sensing image datasets, including UCM, RS19, RSSCN7, and AID, show that for various convolutional neural network architectures, the proposed E-LargeVis can effectively improve retrieval performance, far exceeding other dimensionality reduction methods.

## 1. Introduction

With the rapid development of high-resolution remote sensing and ground observation technology over recent years, the quantity of remote sensing imagery data has increased exponentially. The inability to quickly browse and efficiently find required images from large-scale remote sensing archives has created a bottleneck and causes problems related to remote sensing information management and sharing, which directly influence the utilization of remote sensing data. Content-based image retrieval (CBIR) [1] as a mainstream retrieval solution was proposed in the 1990s and has gradually developed; this has been widely applied in high-resolution remote sensing image retrieval (RSIR). CBIR includes two essential components: feature extraction and similarity measurement, in which image content is represented as image features, and the retrieval results are obtained by measuring feature similarity of images. Therefore, the ability to extract more representative and discriminative image features ultimately determines the performance of CBIR.

In light of the diversity and complexity of image content, the extracted features are usually highly dimensional in order to effectively describe the image content. However, if high-dimensional data are directly applied to image retrieval, a “curse of dimensionality” will occur, so it is difficult for practical application. The reason for the “curse of dimensionality” is that the extreme sparseness of the real data distribution under the high-dimensional situation will lead to a large deviation when measuring the similarity of image features, while causing inefficient feature processing. A fixable and available solution is dimensionality reduction which can project the original high-dimensional space data into a low-dimensional space. For image retrieval, an excellent dimension reduction method can not only effectively remove the redundancy among the data, but also ensure that the retrieval performance will not reduce too much or perhaps even improve. 

Stochastic neighbor embedding (SNE) proposed by Hinton et al. [2] is a probability-based dimension reduction algorithm designed for the visualization of larger-scale high-dimensional data. The basic idea of SNE is that the neighbor relationship should be retained when projecting data points close to each other in high-dimensional space into a low-dimensional space, while the distance of data points is measured by conditional probability. Although SNE can obtain better dimension reduction effects, it is difficult to optimize during training and can easily cause crowding problems. In 2008, Maaten et al. [3] proposed an improved SNE, i.e., *t*-SNE, which replaced the normal distribution with the *t* distribution when calculating the probability of sample points in a low-dimensional space by a symmetric probability calculation to effectively solve the crowding problem. However, the disadvantage of *t*-SNE is its high computational complexity and poor scalability. In 2016, Tang et al. proposed a LargeVis dimensionality reduction algorithm by optimizing *t*-SNE, greatly reducing the computational complexity and allowing more efficient processing of larger-scale high-dimensional data.

Unfortunately, SNE, *t*-SNE or LargeVis cannot achieve low-dimensional data with the feature of a single image because they are designed for the visualization of large-scale high-dimensional data by using the distance relationship of data. Here, an extended LargeVis (E-LargeVis) dimension reduction method is proposed that uses support vector regression (SVR) to fit the LargeVis implicit mapping model between high-dimensional data and low-dimensional data. Using this, we obtain low-dimensional data from high-dimensional data of a single image to satisfy the demands of RSIR.

On this basis, a high-resolution RSIR method is also proposed in this paper. First, the fully connected layer features are extracted by using a channel attention-based ResNet50 as a backbone network. Then, E-LargeVis is used to reduce the dimensionality of the features to obtain a low-dimensional discriminative representation. Finally, the L2 distance is computed as a similarity measurement to realize the retrieval of high-resolution remote sensing images. The experimental results from four high-resolution remote sensing image datasets show that for various convolutional neural network (CNN) architectures, the low-dimensional fully connected layer features reduced by E-LargeVis can effectively remove the redundancy and greatly improve retrieval performance. Compared with the existing high-resolution RSIR methods, the proposed retrieval method can obtain better retrieval performance. 

## 2. Related Works

In this section, we firstly provide a detailed introduction to the dimensionality reduction method, and then comprehensively review the existing research works of high-resolution RSIR based on CNN. 

### 2.1. Dimensionality Reduction Method

Dimensionality reduction is used to map high-dimensional data to low-dimensional data [4]. According to the types of mapping function, the dimensionality reduction algorithms can be divided into linear dimensionality reduction algorithms and nonlinear dimensionality reduction algorithms. Linear dimensionality reduction constructs a linear function based on the sample set to realize the mapping from high-dimensional to low-dimensional space, such as principal components analysis (PCA) [4], linear discriminant analysis (LDA) [5] and multidimensional scaling (MDS) [6], etc., which have been widely used in various image retrieval schemes.

The non-linear dimensionality reduction method conducts dimensionality reduction of high-dimensional data by constructing a nonlinear map, which is adopted to produce a better performance when the data are non-linear. The representative methods are locally linear embedding (LLE) [7], locality preserving projection (LPP) [8], stochastic neighbor embedding (SNE) [2], *t*-distributed stochastic neighbor embedding (*t*-SNE) [3] and LargeVis [9], etc. Among them, SNE, *t*-SNE and LargeVis are designed for the visualization of large-scale high-dimensional data. Zhang et al. [10] utilized a *t*-SNE-based nonlinear manifold hashing algorithm to make dimensionality reduction by learning compact binary codes embedded on the intrinsic manifolds of deep spectral-spatial features for balancing between learning efficiency and retrieval accuracy. 

Compared with *t*-SNE, LargeVis can achieve an equivalent or even better dimensionality reduction effect. *t*-SNE and LargeVis can effectively increase the distance of different classes of data that are far apart in high-dimensional space while decreasing the distance within the class. In addition, the computational complexity of LargeVis is much lower than *t*-SNE. However, there are two problems that need to be solved when applying LargeVis to image retrieval.

1. LargeVis uses the distance relationship of data to reduce dimensionality, and cannot reduce the dimensionality of high-dimensional data of a single image, so it is necessary to extend LargeVis to meet the requirements of image retrieval.
2. LargeVis is unfavorable for image retrieval since it has a high degree of randomness, leading to the different results of dimensionality reduction. It is necessary to eliminate the randomness while taking advantage of the clustering characteristics of LargeVis data.

### 2.2. High-Resolution Remote Sensing Image Retrieval Based on CNN

In recent years, deep learning has made tremendous breakthroughs in many fields such as speech recognition, natural language processing, and computer vision. The most famous deep neural network—convolutional neural network—adopts deep hierarchical architectures with parameters of each layer learned from large labeled classification datasets [11]. Deep features are more robust, discriminative and representative than hand-crafted features, especially for image context information extraction.

**Figure 1:**
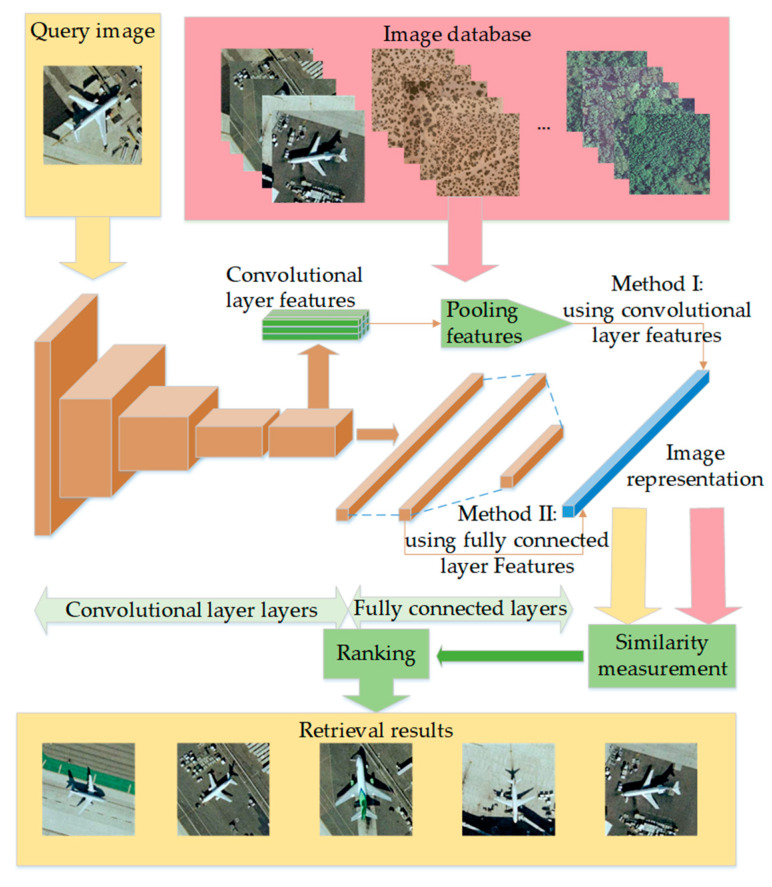
High-resolution remote sensing image retrieval (RSIR) framework based on convolutional neural network (CNN). The query image and image database are represented in the same method. First, the image representation is obtained from convolutional layer features or fully connected layer features. Then, the retrieval results are ranked by similarity measurement.

As mentioned above, CBIR is an image retrieval framework that was proposed in the 1990s, which included image feature extraction and similarity measurement. It is very important for CBIR to extract the representative and discriminative image features [12]. Recently, researchers applied CNN to extract deep features for image retrieval and achieved much better performance than traditional methods [13], which has become a mainstream solution of high-resolution RSIR [14]. The overall framework is shown in Figure 1.

Figure 1 shows that the deep features based on CNN mainly include two types: Convolutional layer features and fully connected layer features. The convolutional layer features contain more details that come from low levels of the CNN, and the fully connected layer features focus more on semantics that come from the high levels of the CNN.

● Convolutional layer features

The output of the CNN convolution layers is feature maps obtained by convolving the image with convolution kernels of various sizes and parameters. Since different convolution kernels have distinctness and diversity in the ability to describe image features, CNN can obtain more abundant image feature representation. In fact, the feature map of the convolutional layer cannot be directly used as an image descriptor, which usually needs to be compactly represented as a descriptor through a coding or pooling operation if it is applied for retrieval or classification.

Zhou et al. [15], Hu et al. [16] and Xia et al. [17] systematically conducted a comparative experiment on retrieval performance by using CNN convolutional layer features and fully connected layer features. In the comparative experiment, AlexNet [11], VGGNet [18], and GoogLeNet [19] were used as CNN backbone networks to extract convolutional layer features. Then, bag-of-words (BoW) [20], improved fisher kernel (IFK) [21] and vector locally aggregated descriptors (VLAD) [22], etc., were used to encode the convolutional layer features. Finally, various pooling methods, including max pooling, average pooling, hybrid pooling, SPoC [23] and CroW [24] were compared and analyzed. The experimental results showed that encoding the convolutional layer features can obtain better retrieval performance than the pooling operation and the convolutional layer features were better than the fully connected layer features.

Wang et al. [25] proposed an image retrieval method based on bilinear pooling, in which the ImageNet dataset [26] was used to pre-train VGG-16 [18] and ResNet34 [27] networks and the convolutional layer features of the two networks were weighted by the channel and spatial attention mechanisms to assign higher weights to useful feature channels for the retrieval task. The deep feature vectors were obtained by fusing the last convolutional layer features of the two networks with bilinear pooling. Finally, PCA was adopted to reduce the dimensionality of the deep features for image retrieval. The experimental results show that this method can achieve better retrieval performance than other pooling methods.

● Fully connected layer features

Fully connected layer features represent the global information of the image, reflecting the semantic information. Napoletano [28] thoroughly explored the impact of network training strategies on retrieval performance and found that the retrieval performance of fully connected layer features on remote sensing images significantly exceeded that of hand-crafted features when only pre-training CNN, and the extracted fully connected layer features achieved optimal performance when using the “pre-training + fine-tuning” CNN training strategy.

However, the dimensionality of the fully connected layer features is often relatively high, leading to the problem of the “curse of dimensionality”. As a result, Xiao et al. [29] proposed a deep compact code (DCC) method to extract low-dimensional CNN features, in which the second fully connected layer of AlexNet and VGGNet networks is utilized to obtain low-dimensional features. Compared with usual CNN features and low-dimensional features obtained by PCA, the low-dimensional features obtained by DCC can effectively improve the performance of RSIR, especially in 64 dimensions.

The fully connected layer features mainly contain semantic information, lacking local details and positional information of the image. Hu et al. [16] and Xia et al. [17] proposed a fully connected layer feature extraction method based on multiple blocks or regions, in which the fully connected layer features of each block are extracted separately to cascade them after dividing the image into blocks. Then, these features were aggregated by using maximum pooling, mean pooling, and mixed pooling, and PCA was used to generate the low-dimensional features for image retrieval. The experimental results show that the fully connected layer features extraction with blocks method can solve the problem of providing positional information. Compared with the method of extracting fully connected layer features from the whole image, it can effectively improve retrieval performance.

Li et al. [30] proposed a feature extraction method for fully connected layers based on regions of interest (ROIs). First, the ROIs of the image were determined. Then, the fully connected layer features of the ROIs were extracted and further encoded by VLAD; finally, PCA was used to reduce the dimensionality of the features. The experimental results show that this method can obtain higher retrieval performance than the methods of extracting fully connected layer features from the whole image.

Another core part of CBIR is similarity measurement, in which distance measurement is the most commonly used method. Until now, many researchers have carried out the work on learning-based distance measurement. The learning-based distance measurement method is to learn embedding space so that the distance of similar features is closer, and the distance of dissimilar features is further away. Ye et al. [31] used the similarity of image classes to sort the CNN feature distance of the query image and each retrieved image in ascending order to obtain the initial retrieval result. Then, the initial retrieval results are reordered by a weight, which is calculated from the query image and each class according to the initial retrieval results. The retrieval performance is superior to the state-of-the-art methods. Cao et al. [32] proposed a triple network that outputs the feature vectors of images, positive and negative samples and normalizes them. Finally, the distance of feature vectors is used to calculate the loss value, to get closer to the positive samples and further from the negative samples. The final retrieval performance is significantly better than the existing methods. Moreover, Zhang et al. [33] introduced correlation feedback based on feature weighting to further improve retrieval accuracy.

## 3. The Proposed E-LargeVis Method

In this section, we first review the principle of the LargeVis dimensionality reduction method and then introduce the extended LargeVis method in our work.

### 3.1. Principle of LargeVis Dimensionality Reduction Method

Although *t*-SNE and its improved algorithms have been widely used, there are two shortcomings: (1) When processing large-scale high-dimensional data, *t*-SNE has low computational efficiency (including its improved algorithms); (2) *t*-SNE has a poor pervasive, for example, it cannot be applied to other datasets when adjusting the parameters on one dataset, and it takes a lot of time to re-adjust the parameters. Tang et al. [9] improved the *t*-SNE and proposed LargeVis dimensionality reduction algorithm. The main improvement points include an efficient kNN graph construction algorithm, a low-dimensional space visualization algorithm and an objective function. 

Constructing an accurate kNN graph requires extremely high computation complexity because the distance between two data points needs to be calculated. LargeVis adopts a neighbor search method, which is divided into two steps. (1) After the space is divided by using a random projection tree, the *k* nearest neighbors of each point are found to obtain an initial kNN graph that does not require complete accuracy so as to speed up the calculation of the probability value of the sample point. (2) The potential neighbors are found using the neighbor search algorithm; then, the distance of the neighbor and the current point, and the neighbor’s neighbor and the current point are calculated to put into the root pile, in which the *k* nodes with the smallest distance are taken as *k* nearest neighbors; finally, an accurate kNN graph can be obtained.

Since LargeVis regards the current center point as the target in the original high-dimensional space, in which the center point and its neighbor nodes constitute a positive sample, and the center point and non-neighbor points constitute a negative sample. The weight of positive samples is the same as that defined in *t*-SNE. The conditional probability from data ${\vec{x}}_{i}$ to ${\vec{x}}_{j}$ is first calculated as:

(1) $$p_{j|i}=\frac{\exp\left(-\|{\vec{x}}_{i}-{\vec{x}}_{j}\|{}^{2}/2\sigma_{i}^{2}\right)}{\sum_{\left(i,k\right)\in E}\exp\left(-\|{\vec{x}}_{i}-{\vec{x}}_{k}\|{}^{2}/2\sigma_{i}^{2}\right)},p_{i|i}=0$$

where $\sigma_{i}$ is selected by setting the complexity of the conditional distribution $p_{⬝|i}$ equal to complexity *u*. Then the graph is symmetrized by setting the weight between ${\vec{x}}_{i}$ and ${\vec{x}}_{j}$ as:

(2) $$\omega_{ij}=\frac{p_{j|i}+p_{i|j}}{2N}$$

In the low-dimensional space, the coordinate position is determined by the probability of observation. We first define the probability of observing a binary edge $e_{ij}=1$ between a pair of vertices as follows:

(3) $$P\left(e_{ij}=1\right)=f\left(\|{\vec{y}}_{i}-{\vec{y}}_{j}\|\right)$$

where ${\vec{y}}_{i}$ and ${\vec{y}}_{j}$ are the embedding of the pair of vertices in the low-dimensional space, *f* (·) is a probabilistic function with respect to the distance of vertex ${\vec{y}}_{i}$ and ${\vec{y}}_{j}$, i.e.,$\;\|{\vec{y}}_{i}-{\vec{y}}_{j}\|$. When the two vertices are close in the low-dimensional space, there is a high probability of finding a binary edge between the two vertices. To further extend to general weighted edges, the possibility of observing a weighted edge $e_{ij}=\omega_{ij}$ is defined as follows:

(4) $$P\left(e_{ij}=\omega_{ij}\right)=P{\left(e_{ij}=1\right)}^{\omega_{ij}}$$

According to the above definition, given a weighted graph *G =* (*V,E*), the possibility of the graph can be calculated as:

(5) $$O=\underset{\left(i,j\right)\in E}{\prod }p{\left(e_{ij}=1\right)}^{\omega_{ij}}\underset{\left(i,j\right)\in \bar{E}}{\prod }{\left(1-p\left(e_{ij}=1\right)\right)}^{\gamma }\;\propto \sum_{\left(i,j\right)\in E}\omega_{ij}\log p\left(e_{ij}=1\right)+\sum_{\left(i,j\right)\in \bar{E}}\gamma \log\left(1-p\left(e_{ij}=1\right)\right)$$

where *E* is the set of unobserved vertex pairs and a unified weight is assigned to the negative edges. The first part represents the possibility of the observed edges and will remain close together in the low-dimensional space by maximizing the similar data points. The second part represents the possibility of other vertex pairs without edges. Data from different classes will be further away from each other by maximizing the second part. By maximizing the equation, both goals can be achieved.

According to the above objective function, optimization requires a lot of computational overhead because the number of negative edges is large, and directly training all negative edges will cause a further rise in complexity. Therefore, the LargeVis algorithm uses a negative sampling algorithm for optimization. For each vertex *i*, some vertices *j* are sampled randomly according to a noisy distribution $P_{n}\left(j\right)$ and treat (*i, j*) as the negative edges, in which the probability meets the noise distribution as:

(6) $$P_{n}\left(j\right)\propto d_{j}^{0.75}$$

where $d_{j}$ is the degree of *j* and *M* is the number of negative samples for each positive edge. The function can be redefined as:

(7) $$O=\sum_{\left(i,j\right)\in E}\omega_{ij}\log p\left(e_{ij}=1\right)+\sum_{k=1}^{M}E_{jk}\sim P_{n}\left(j\right)\gamma \log\left(1-p\left(e_{jk}=1\right)\right)$$

After using negative sampling and edge sampling optimization, LargeVis also uses the asynchronous stochastic gradient descent method for training, which effectively reduces the computation complexity of the algorithm. 

### 3.2. Extended LargeVis Method

As mentioned above, the LargeVis algorithm is designed for the visualization of large-scale high-dimensional data. The distance of each point is essential so that LargeVis cannot achieve dimensionality reduction for high-dimensional data of a single image. Hence, the LargeVis algorithm is expanded by SVR to fit the implicit mapping function for reducing the dimensionality of high-dimensional data of a single image.

**Figure 2:**
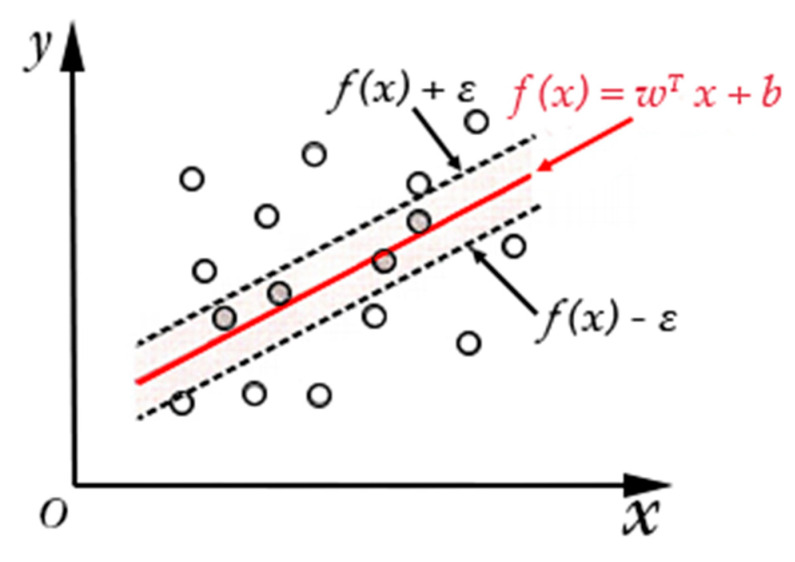
Principle of Support Vector Regression (SVR).

Support vector regression (SVR) is an effective multiple regression method founded on the support vector machine, which can regress nonlinear problems well. Its goal is to find an optimal hyperplane and control the error between all training samples and the optimal hyperplane to achieve the regression analysis, as shown in Figure 2, in which ε is the fitting accuracy control parameter.

With SVR, as long as the value inside the dotted line can be regarded as a correct prediction, only the loss of the value outside the dotted line is calculated. The optimization problem of SVM regression is obtained by introducing the relaxation variables $\xi_{i}$ ≥ 0 and $\xi_{i}^{*}$ ≥ 0:

(8) $$min_{\left(w,b,\xi \right)}\frac{1}{2}{\left\|w\right\|}^{2}+C\sum_{i=1}^{n}\xi_{i}+\xi_{i}^{*}$$

(9) $$y_{i}-w^{T}x_{i}-b\leq \varepsilon +\xi_{i}$$

(10) $$w^{T}x_{i}+b-y_{i}\leq \varepsilon +\xi_{i}^{*}$$

(11) $$\xi_{i}\geq 0,\;\xi_{i}^{*}\geq 0,i=1,2,3\ldots n$$

The Lagrange multiplier is introduced to obtain the linear fitting function as:

(12) $$f\left(x\right)=w^{T}x_{i}+b=\sum_{i=1}^{n}\left(\alpha_{i}-\alpha_{i}^{*}\right)\left(x.x_{i}\right)+b$$

where $\alpha_{i}$ and $\alpha_{i}^{*}$ are Lagrange’s multipliers. The above function can be obtained by adding a kernel function as:

(13) $$f\left(x\right)=w^{T}x_{i}+b=\sum_{i=1}^{n}\left(\alpha_{i}-\alpha_{i}^{*}\right)K\left(x_{i},x\right)+b$$

**Figure 3:**
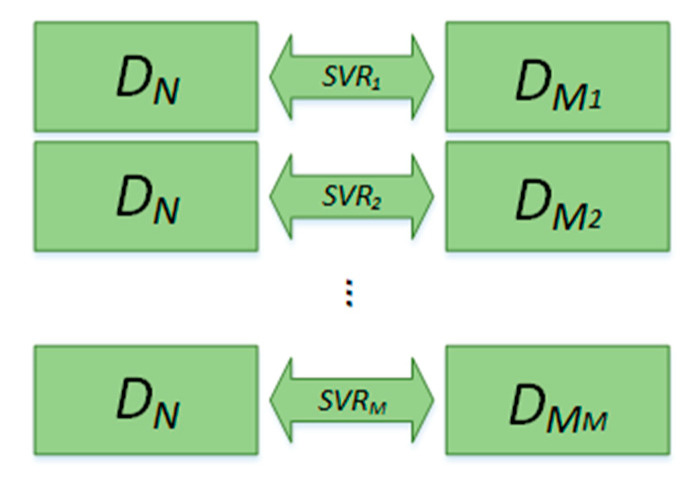
Principle of LargeVis training-based SVR.

Compared with other fitting methods, SVR can produce optimal fitting accuracy. Therefore, we used SVR as the fitting method. As can be seen in Figure 3, fitting is divided into a training phase and a dimensionality reduction phase, in which the training phase is performed offline. The specific process is as follows:

Training phase:

1. Low-dimensional data of all training sets are obtained by LargeVis. It is necessary to reduce the high-dimensional data to a different dimensionality to meet different requirements. The sample pairs of training data are composed of high-dimensional and low-dimensional data, in the form of $D_{N}-\mathrm{LargeVis}-D_{M}$, $D_{M}=\left[D_{M_{1}},D_{M_{2}},\ldots ,\;D_{M_{M}}\;\right]$, where $D_{N}$ is high-dimensional training data with *N* dimensionality and $D_{M}\;$ is low-dimensional training data with *M* dimensionality, $D_{M_{1}}$ is the first dimension of $D_{M}$, and $D_{M_{M}}$ is the *M*th dimension of $D_{M}$.
2. The sample pairs are input into the SVR to build a mapping model of high-dimensional data to low-dimensional data. In particular, each component of low-dimensional data needs one SVR fitting model.

**Figure 4:**
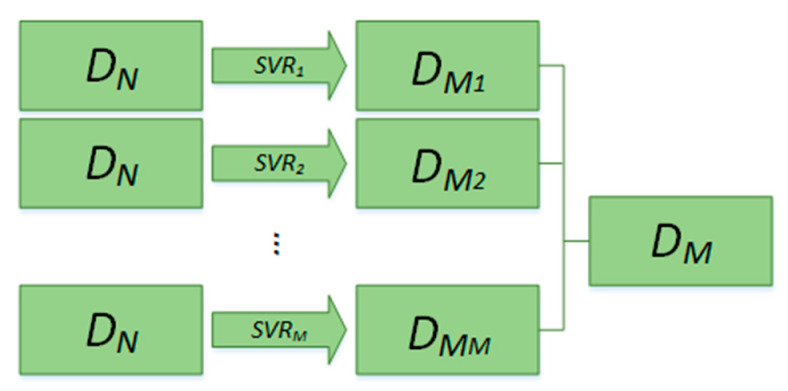
Principle of dimensionality reduction of LargeVis-based SVR. The low-dimensional data are obtained by combining the results of SVR in order.

Dimensionality reduction phase:

The appropriate SVR fitting model is selected according to the dimensionality reduction demands. The final dimensionality reduction data can be obtained by combining all fitting results in order, as shown in Figure 4.

## 4. High-Resolution Remote Sensing Image Retrieval Method

**Figure 5:**
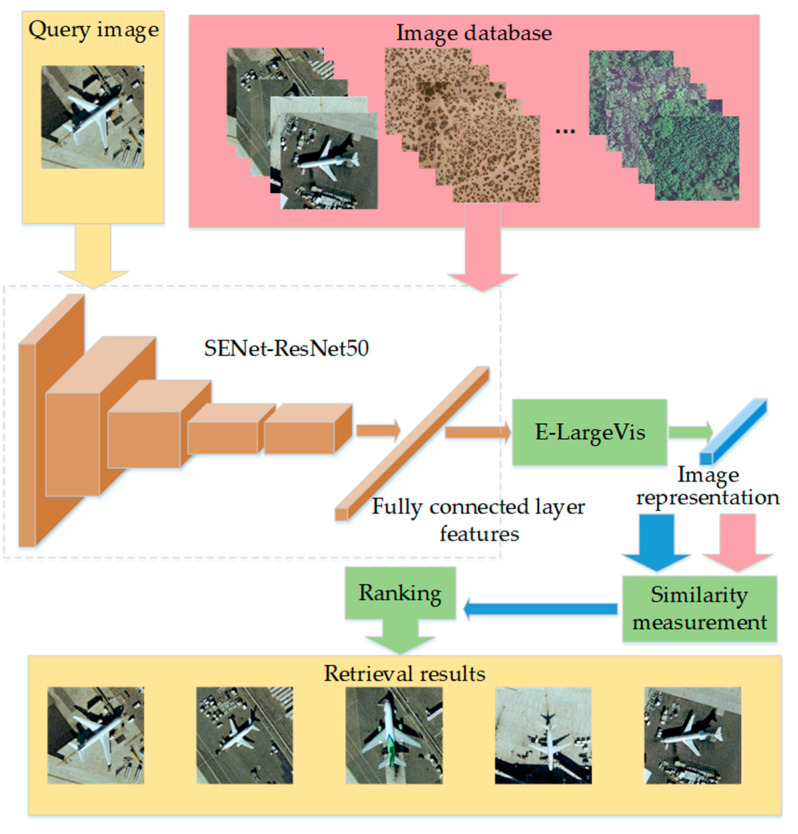
Channel attention-based high-resolution RSIR. Firstly, the fully connected layer features are extracted by SENet-ResNet50. Then, Extended LargeVis (E-LargeVis) was used to reduce the dimensionality of the features to obtain a low-dimensional discriminative representation. Finally, L2 distance was computed to measure similarity and realize the retrieval of high-resolution remote sensing images.

In this section, we apply the proposed E-LargeVis method to high-resolution remote sensing image retrieval. The overall process is shown in Figure 5, in which the fully connected layer features are extracted by using a channel attention-based ResNet50 as a backbone network. Then, E-LargeVis is used to reduce the dimensionality of the features to obtain a low-dimensional discriminative representation. Finally, L2 distance is computed for similarity measurement to realize the retrieval of high-resolution remote sensing images. 

### 4.1. Channel Attention-Based ResNet50

In this section, we firstly make a detailed introduction to ResNet network architecture and then review the channel attention mechanism. 

#### 4.1.1. ResNet50 Network

The ResNet network was proposed by He et al. [27] in 2015. Its main contribution is to solve the problem that the classification accuracy decreases with the deepening of the CNN. Besides that, the proposed residual learning idea accelerates the CNN training process, which effectively avoids the problem of vanishing gradient and explosion.

**Figure 6:**
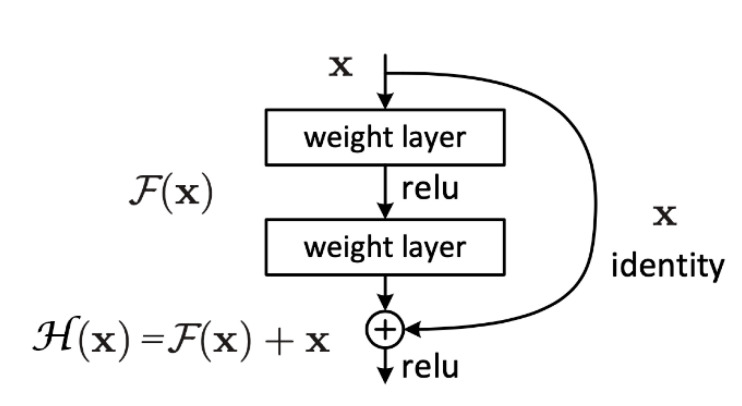
Schematic diagram of shortcut connection [27].

Driven by the idea of residual learning, He et al. proposed a shortcut connection structure of identity mapping, as shown in Figure 6, where *x* is the input, *H*(*x*) is the desired underlying mapping, *F*(*x*) is residual mapping, and *H*(*x*) *= F*(*x*) *+ x*. By transforming the network from fitting desired underlying mapping *H*(*x*) into fitting the residual mapping *F*(*x*), the output can be turned into a composition of the input and residual maps, making the network more sensitive to change between input *x* and output *H*(*x*).

**Figure 7:**
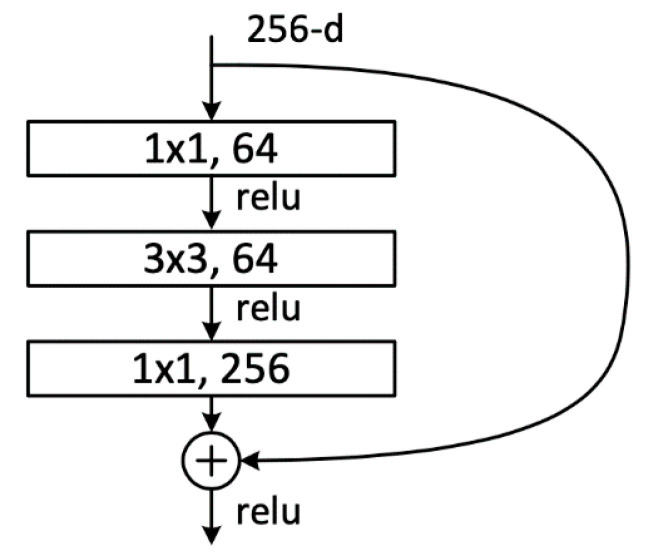
Schematic diagram of bottleneck [27].

To build a deeper network structure, He et al. [27] also conducted the bottleneck structure, as shown in Figure 7. To adapt to the deeper network structure, the bottleneck structure adds a 1 × 1 convolution to reduce the input dimensionality. The bottleneck structure is used in ResNet-50/101/152 networks.

Lately, ResNet has been widely applied in various computer vision tasks, and has achieved outstanding performance. In this paper, ResNet50 was selected as the backbone network to extract the fully connected layer features of the image for image retrieval.

#### 4.1.2. Channel Attention Mechanism

**Figure 8:**
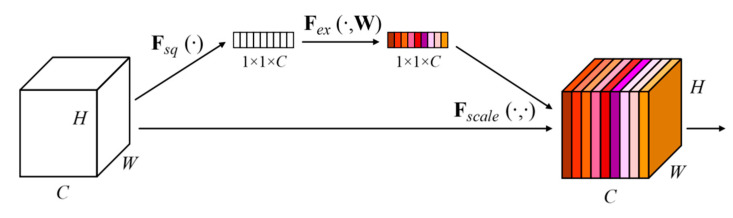
Schematic diagram of SENet block [34]. The size of the original feature map was *H × W × C*, where H is the height, W is the width, and C is the number of channels. *Fsq*(·) compressed the feature map from *H × W × C* to 1 × 1 × *C*; then *Fex*(·,*W*) learns the dependence of each channel and *Fscale*(·, ·) adjusts the feature map according to the dependence.

In the general structure of CNN, the convolutional layer does not take into account the dependence of the output results and the channel features. The basic idea of the attention mechanism is to allow the network to selectively enhance features with more contribution to the task as well as suppress unimportant features, in which the channel attention mechanism is one of the most commonly used attention mechanisms. The squeeze-and-excitation networks (SENet) proposed by Hu et al. [34] in 2017 is one of the representative works. The SENet block is shown in Figure 8.

The channel attention mechanism is divided into three parts: Squeeze, excitation, and scale. First, squeeze is used to encode the feature map in the channel as a global feature, which can be implemented by global pooling, where $u_{c}$ represents the *C*th convolution kernel in the convolution layer, and *H* and *W* represent the size of the convolution kernel:

(14) $$z_{c}=F_{\mathrm{squeeze}}\left(u_{c}\right)=\frac{1}{H\times W}\sum_{i=1}^{H}\;\sum_{j=1}^{W}u_{c}\left(i,j\right)$$

After the squeeze operation, a global feature description is obtained. Then, the global description features are passed through an excitation function to learn the nonlinear relationship of the channels. The incentive mechanism in the form of Sigmoid is adopted as:

(15) $$s=F_{\mathrm{excitation}}\left(z,W\right)=\sigma \left(g\left(z,W\right)\right)=\sigma \left(W_{2}\mathrm{RELU}\left(W_{1}z\right)\right)$$

where $W_{1}\in R^{\frac{C}{r}\times C}$, $W_{2}\in R^{C\times \frac{C}{r}}$. We used two fully connected layer structures. The first fully connected layer structure can reduce the dimensionality to *r* dimensionality (*r* is a hyper-parameter). Here, dimensionality reduction can decrease the computational complexity of the model. The second full connection restores the dimensionality to the *C* dimensionality and aligns with the original number of convolution kernels. Finally, the activation value is multiplied correspondingly to the original feature channel as:

(16) $$\tilde{x}=F_{\mathrm{scale}}\left(u_{c},s_{c}\right)=s_{c}\cdot u_{c}$$

Except for the hyper-parameter *r* in the entire calculation process, the remaining parameters are learned during the training process. The hyper-parameter *r* allows us to vary the capacity and computational cost in the network. Hu et al. [34] conducted experiments for a range of different *r* values. Setting *r* = 16 achieves a good balance between accuracy and complexity. In the learning process, the more useful feature channels for the task will be assigned a higher weight, which means that the representation ability of these “important features” has been enhanced. This structure can be integrated into many existing networks, such as Inception, ResNet, etc.

**Figure 9:**
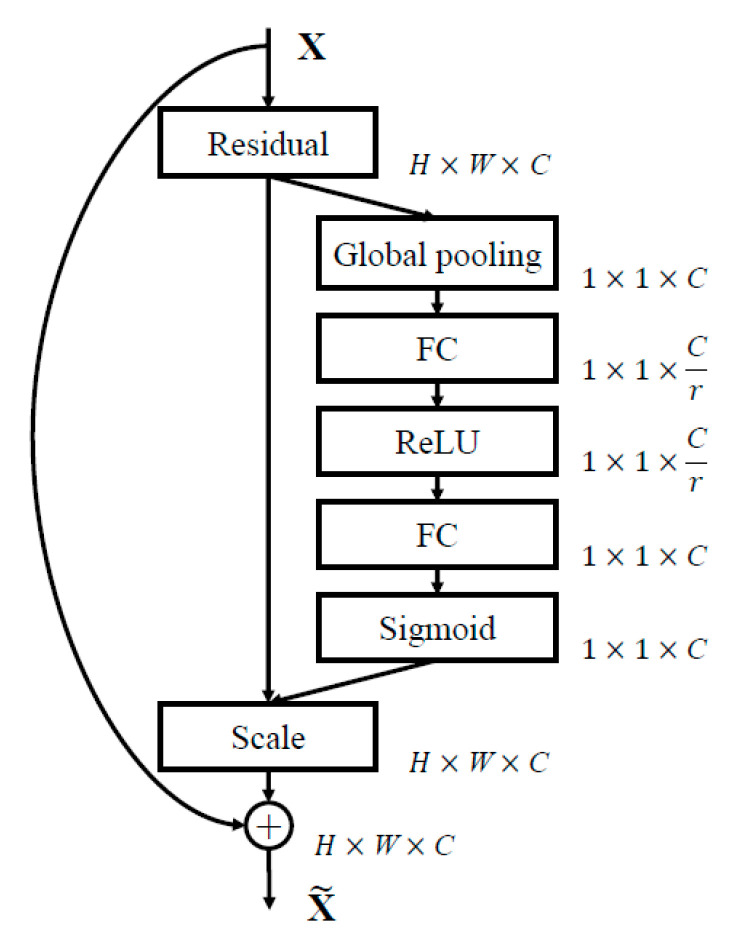
Channel attention-based ResNet residual module [34]. The global pooling layer and fully connected layers were added to the residual module to calculate the excitation parameters, and the features with channel attention were obtained from multiplied original features with excitation parameters.

The channel attention structure is easy to implement. We integrated the channel attention structure into the ResNet50 network as a backbone network to extract features, named the SENet-ResNet50 network. The SENet-ResNet50 network residual module is shown in Figure 9.

In the embedding mode, the squeeze and excitation operations are performed on the ResNet residual module, and then the processed residual module is added to the identity map *x* as the output of this layer.

In this paper, first, the fully connected layer features were extracted from SENet-ResNet50, and then E-LargeVis was used to reduce the dimensionality of the features to avoid the “curse of dimensionality”. Finally, the obtained low-dimensional features were utilized to perform similarity measurement.

### 4.2. Similarity Measurement

The Euclidean distance of feature vectors is adopted to measure the similarity of images, which is defined in Euclidean space. The Euclidean distance between two points $x_{1k}$ (*k* = 1, 2 … *n*) and $x_{2k}$ (*k* = 1, 2 … *n*) in *N* dimensional space is defined as follows:

(17) $$d_{12}=\sqrt{\sum_{k=1}^{n}{\left(x_{1k}-x_{2k}\right)}^{2}}$$

## 5. Experimental Results and Analysis

To evaluate the performance of the proposed E-LargeVis dimensionality reduction method and the proposed high-resolution RSIR method of a channel attention-based ResNet50, we made comparisons with four high-resolution remote sensing image datasets including UCM, RS19, RSSCN7, and AID.

### 5.1. Datasets and Evaluation Metric

The changes in the surface of the Earth is usually a long-term developing period, and require high resolution in order to be properly recognized and retrieved [35]. UCM, WHU-RS, RSSCN7 and AID are currently the four most commonly used high-resolution remote sensing image datasets.

The images in the UCM dataset [36] come from the United States Geological Survey’s city map, which contains a total of 21 categories including airplanes, beaches, buildings, and dense residential areas. Each category contains 100 images of 256 × 256 size, and the spatial resolution of each pixel is 0.3 m.

The WHU-RS19 dataset [37] is a remote sensing image dataset released by Wuhan University in 2011. The image size is 600 × 600, and it contains 19 types of scene images. Each type contains about 50 images, for a total of 1005 images.

The RSSCN7 dataset [38] is a remote sensing image dataset released by Wuhan University in 2015 and contains a total of 2800 images. These images come from seven typical scenes including grassland, forest, farmland, parking lot, residential area, industrial area and lake. Each category includes 400 images, which were collected from Google Maps during different seasons and weather changes, corresponding to four scales of sampling, each scale with 100 images with a size of 400 × 400. Due to the variety of scenarios, this dataset presents some challenges.

The AID dataset [39] is a remote sensing image dataset jointly released by Wuhan University and Huazhong University of Science and Technology in 2017 containing 30 types of scenes, each type containing 220–420 images. There is a total of 10,000 images, each sized 600 × 600.

We used mean average precision (mAP) [40] to evaluate retrieval performance, which is the accepted image retrieval performance evaluation index. The mAP is the mean value of average precisions of a set of queries, and it measures the average retrieval precision across all the query images. The mAP is defined as follows:

(18) $$\mathrm{mAP}=\frac{\sum_{q=1}^{Q}AveP\left(q\right)}{Q}$$

The definition of *AveP* is:

(19) $$AveP=\frac{\sum_{k=1}^{n}\left(P\left(k\right)\times rel\left(k\right)\right)}{number\;of\;relevant\;images}$$

where *P*(*k*) is the accuracy rate and *rel*(*k*) is a piecewise function. When the *k*th image is a related image, its value is 1, otherwise, it is 0.

### 5.2. Experimental Setting

In our experiments, the dataset was randomly divided and experiments were repeated five times, with the final results being the average. In the experiment, 80% of the images were randomly selected from each dataset as training samples, and the remaining 20% of the images were used as test samples. The training samples were expanded by rotating the original image and its horizontal mirror image once every 45°. The expanded dataset was 16 times the original size; this was used to train the CNN network model.

Our network was built and tested in the Keras open-source framework. The experimental platform uses Intel Core i7-8700 (Intel Corporation, Santa Clara, CA, USA), CPU 3.2 GHz, 32 GB memory, and contains an NVIDIA GeForce RTX 2080 Ti graphics card (NVIDIA Corporation, Santa Clara, CA, USA) for training and testing. The number of training iterations was set to 50 rounds, the batch size was set to 16, and the learning rate was 0.01. Momentum and weight decay methods were used to optimize the training process to prevent overfitting. The weight decay rate was 0.0001, and the momentum parameter was set to 0.9.

### 5.3. Experiment I: Performance Comparison of Different CNN

To verify the robustness and representation of the deep features extracted by different CNN architectures, we used AlexNet [11], VGG-16 [18], GoogLeNet [19], ResNet50 [26] and SENet-ResNet50 networks to extract fully connected layer features. In the experiment, for each CNN, the ImageNet dataset [26] was used for pre-training to obtain the initial parameters of the network model, and then the high-resolution remote sensing image dataset was used to fine-tune the initial parameters to obtain the network model. The fully connected layers of the network were extracted as deep features for image retrieval. Table 1 shows the dimensionality of the fully connected layer features extracted by the five CNN architectures.

Table 2 shows the comparison results of mAP with different CNN networks on four datasets.

It can be seen from Table 2 that our SENet-ResNet50 can achieve a significant improvement in retrieval performance compared with the other four network architectures on different datasets. The mAP of SENet-ResNet50 was 96.64%, 97.69%, 85.10% and 89.03%, and the largest improvements compared to other methods were as follows: 33.75% higher than VGG-16 in the UCM dataset, 27.26% higher than GoogLeNet in the WHU-RS dataset, 10.88% higher than ResNet50 in the RSSCN7 dataset, and 25.52% higher than GoogLeNet in the AID dataset. This shows that the deep features extracted by the SENet-ResNet50 network architecture have a stronger representative and discriminative ability. In addition, compared with ResNet50, the mAP of SENet-ResNet50 increased from 93.76% to 97.69% for the WHU-RS dataset, and even from 74.22% to 85.10% for the RSSCN7 dataset, which shows that the channel attention mechanism can further improve the representation ability of ResNet50 deep features, thereby improving the retrieval performance.

### 5.4. Experiment II: Performance Comparison of SVR Regression Method

To verify the performance of SVR, we performed regression on the result of LargeVis by SVR, Ridge Regression and Lasso. The parameter kernel of SVR was set to “rbf”, degree of the polynomial kernel function was set to 3 and the parameter gamma of SVR was set to “auto”. The parameter cv of Ridge Regression and Lasso were set to the optimalresult from the –5 power of 10 to the 2 power of 10 with a length of 10, 20 and 30. The retrieval experimental comparison results of SVR and other regression methods were shown in Table 3.

It can be seen from Table 3, SVR obtains better performance in all four datasets and dimensions. Ridge Regression and Lasso are classic regression methods, which are widely used for data regression. In this experiment, SVR is at least 0.58% higher than other methods in mAP of image retrieval. Since the results are from the regression of LargeVis, the retrieval performance is affected by the performance of CNN, LargeVis and regression. Therefore, SVR is chosen as the regression method in this paper.

### 5.5. Experiment III: Performance Comparison of E-LargeVis Dimensionality Reduction

**Figure 10:**
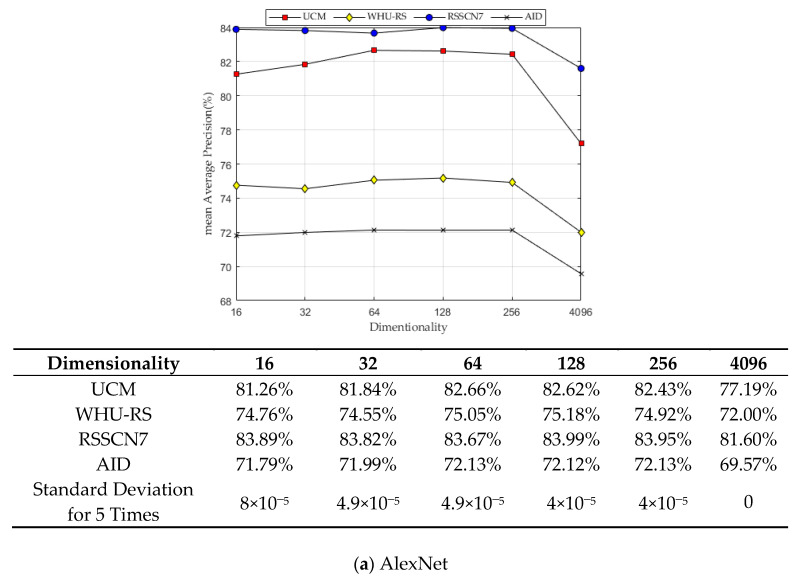

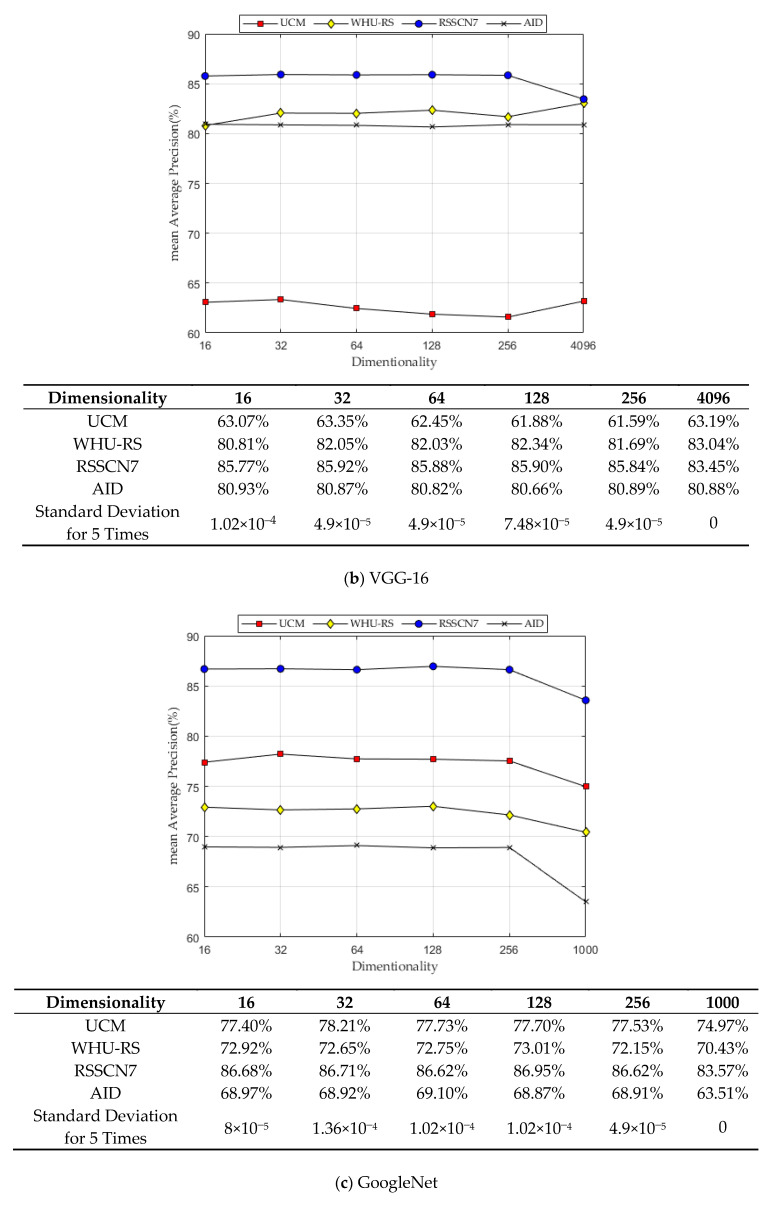

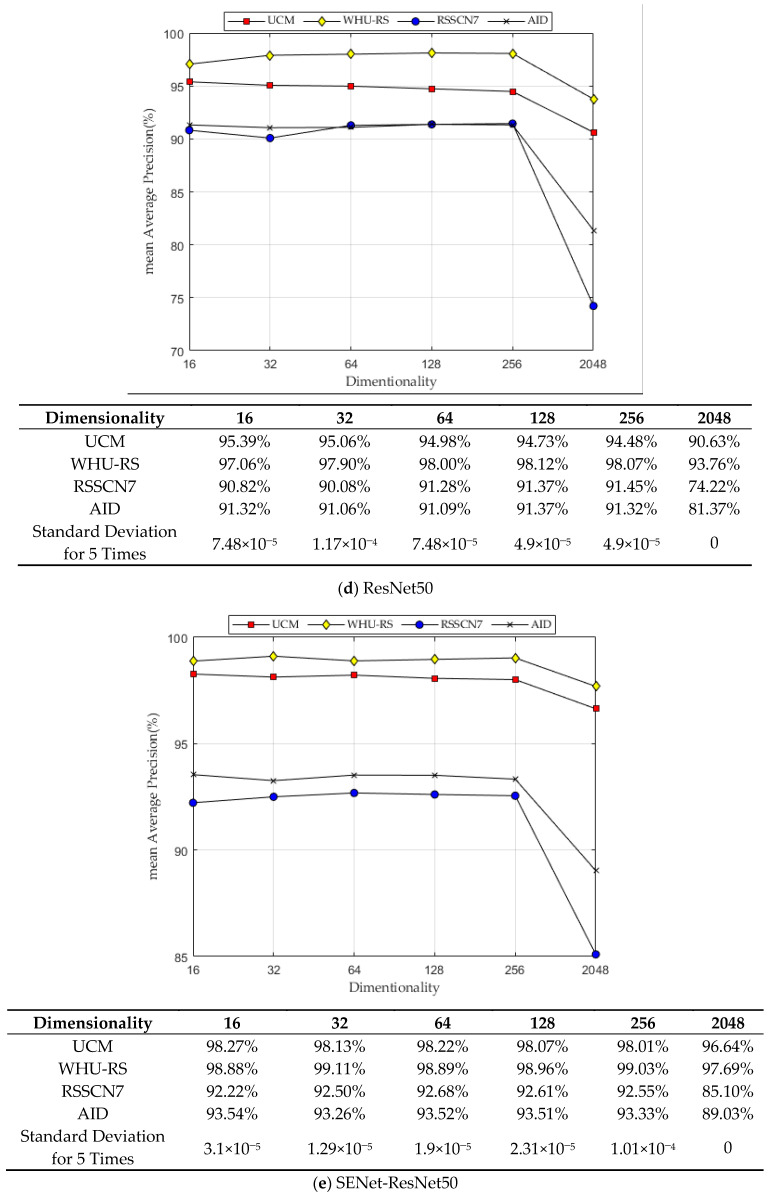
E-LargeVis dimensionality reduction results of different CNN. (**a**) The dimensionality reduction results of AlexNet. (**b**) The dimensionality reduction results of VGG-16. (**c**) The dimensionality reduction results of GoogLeNet. (**d**) The dimensionality reduction results of ResNet50. (**e**) The dimensionality reduction results of SENet-ResNet50.

To verify the performance of the proposed E-LargeVis dimensionality reduction method, we performed dimensionality reduction on the deep features extracted with five CNN. Figure 10 shows the comparison results of mAP in different dimensionality using the E-LargeVis dimensionality reduction method.

It can be seen from the experimental results that only the retrieval performance for the WHU-RS dataset was lower after using E-LargeVis, in which the maximum reduction was from 83.04% to 80.81% when compared with the VGG-16 network. In all other cases, using E-LargeVis improved the retrieval performance, with the largest increase from 74.22% to 91.45% (ResNet50 network with the RSSCN7 dataset). LargeVis can increase the distance of clusters that are far apart in the high-dimensional space after dimensionality reduction, so the low-dimensional features have a stronger discriminative ability for improving retrieval performance.

Through a comprehensive comparison of the retrieval performance under various dimensionalities, the proposed retrieval scheme can reach optimal performance with 64 dimensions considering the dimension as low as possible. Therefore, we set the dimensionality of E-LargeVis to 64.

### 5.6. Experiment IV: Performance Comparison of Euclidean and Other Similarity Measurement Methods

To verify the performance of the Euclidean distance, we also compared it with the other classical similarity measurement methods such as Cityblock, Chebychev, Cosine, Correlation and Spearman. The experimental comparison results of Euclidean distance and other similarity measurement methods are shown in Table 4.

It can be seen from Table 4 that the Euclidean distance obtains better performance in most cases. The result with the greatest difference is Correlation with 16 dimensions in the RSSCN7 dataset which is 92.41%, and is 0.21% higher than the Euclidean distance method. Considering all the results, the Euclidean distance is chosen as the similarity measurement method in this paper.

### 5.7. Experiment V: Performance Comparison of E-LargeVis and Other Dimensionality Reduction Methods

PCA is the most representative linear dimensionality reduction method, while LPP and LLE are the two representative nonlinear dimensionality reduction methods. To verify the performance of the E-LargeVis method, we also compared it with the other classical dimensionality reduction methods such as PCA [4], LPP [8], and LLE [7], in which the CNN architecture uses SENet-ResNet50. The number of neighbors of LPP and LLE was set to the optimal result of 12, 32, 64, 128 and 256. The experimental comparison results of E-LargeVis and other dimensionality reduction methods are shown in Table 5.

It can be seen from Table 5 that the mAP of LPP method was 98.92%, which was 0.04% higher than E-LargeVis with 16 dimensions in the WHU-RS dataset. In all other cases, E-LargeVis can obtain much better retrieval performance. PCA is a linear dimensionality reduction method, so when dimensionality reduction is performed on nonlinear and high-dimensional deep features, PCA fails to obtain the best performance. Although LPP and LLE are also nonlinear dimensionality reduction methods, the performance of these two methods depends on the number of samples and the parameters. If the optimal configuration of parameters cannot be achieved, it is difficult to obtain good retrieval performance using these methods.

### 5.8. Experiment VI: Image Retrieval Results

**Figure 11:**
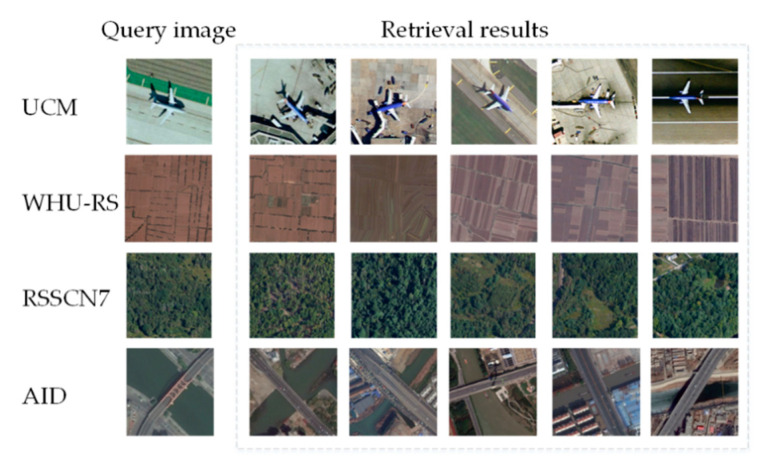
Top five image retrieval results.

The retrieval results of remote sensing images are shown in Figure 11. From the retrieval results, our method obtained better retrieval results compared to other methods, and the similarity ranking also roughly conforms to the human visual system. The images with high similarity to the query image rank first in the retrieval results.

### 5.9. Experiment VII: Performance Comparison with the Existing Methods

To verify the effectiveness of our method, we compared it with the existing nine advanced high-resolution RSIR methods. The retrieval performance comparison results using four datasets are shown in Table 6, in which the experimental results of the other methods in the table are referenced from the literature.

As can be seen from Table 6, our method obtained the optimal retrieval performance for all four datasets. For the most challenging RSSCN7 dataset, our method achieved a mAP of 92.68%. Our method adopts the SENet-ResNet50 network, which can obtain deep features with discriminative representation. Using E-LargeVis to reduce the dimensionality of the deep features not only reduces the computation complexity and saves storage space, but can further enhance the discriminative ability of deep features so as to obtain optimal retrieval performance.

## 6. Discussion

In this paper, an extended LargeVis (E-LargeVis) dimensionality reduction method for high-resolution RSIR was proposed, which can realize the dimensionality reduction of high-dimensional data of single image by modeling the implicit mapping relationship between LargeVis high-dimensional data and low-dimensional data with SVR. Then, we proposed a high-resolution RSIR method by using channel attention and E-LargeVis. First, the fully connected layer features were extracted by using a channel attention-based ResNet50 as a backbone network. Then, E-LargeVis was used to reduce the dimensionality of the features to obtain a low-dimensional discriminative representation. Finally, L2 distance was computed for similarity measurement to realize the retrieval of high-resolution remote sensing images. 

Seven experiments were conducted to verify the effectiveness of our method. In Experiment I, AlexNet, VGG-16, GoogLeNet, ResNet50 and SENet-ResNet50 networks were used to extract FC layer features. For each CNN network, the ImageNet dataset was used for pre-training to obtain the initial parameters of the network model, and then the high-resolution remote sensing image dataset was used to fine-tune the initial parameters to obtain the network model. The number of training iterations was set to 50 rounds, the batch size was set to 16, and the learning rate was 0.01. Momentum and weight decay methods were used to optimize the training process to prevent overfitting. The weight decay rate was 0.0001, and the momentum parameter was set to 0.9. It can be seen from Table 2 that SENet-ResNet50 can achieve a significant improvement in retrieval performance compared with the other four networks on all four datasets. In Experiment II, we performed regression on the result of LargeVis by SVR, Ridge Regression and Lasso. The parameter kernel of SVR was set to “rbf”, degree of the polynomial kernel function was set to 3 and the parameter gamma of SVR was set to “auto”. The parameter cv of Ridge Regression and Lasso were set to the optimal result from the –5 power of 10 to the 2 power of 10 with a length of 10, 20 and 30. As can be seen in Table 3, SVR obtained better performance in all four datasets and dimensions, which was at least to 0.58% higher than other methods in mAP of image retrieval. In Experiment III, the E-LargeVis dimensionality reduction method was performed on the deep features extracted with five CNN. The dimension reduction was set to 16, 32, 64, 128 and 256. It can be seen from Figure 10 that the retrieval performance with the WHU-RS dataset dropped after using E-LargeVis, whereas it improved for all other cases. LargeVis can increase the distance of clusters that are far apart in the high-dimensional space after dimensionality reduction, so the low-dimensional features have a stronger discriminative ability to improve retrieval performance. In Experiment IV, we verified the performance of the Euclidean distance. Compared with the other classical similarity measurement methods such as Cityblock, Chebychev, Cosine, Correlation and Spearman, the Euclidean distance obtained better optimal in most cases. In Experiment V, PCA, LPP, LLE and E-LargeVis ware used to reduce the dimensionality of deep features of SENet-ResNet50. The number of neighbors of LPP and LLE was set to the optimal result of 12, 32, 64, 128 and 256. It can be seen from Table 5 that the mAP of LPP method was 98.92% which was 0.04% higher than E-LargeVis with 16 dimensions in WHU-RS dataset; however, E-LargeVis can obtain better retrieval performance for all other cases. PCA is a linear dimensionality reduction method, so when dimensionality reduction is performed on nonlinear and high-dimensional deep features, PCA fails to reach the best performance. Although LPP and LLE are also nonlinear dimensionality reduction methods, the performance of these two methods depends on the number of samples and the parameters. If the optimal configuration of parameters cannot be achieved, it is difficult to obtain available retrieval performance. In Experiment VI, the top five images from our retrieval method were shown. Our method obtained the best retrieval results, and the similarity ranking also roughly conformed to the human visual system. The images with high similarity to the query image ranked first in our retrieval results. In Experiment VII, we compared our method with the existing nine advanced high-resolution RSIR methods. As can be seen from Table 6, our method obtained the optimal retrieval performance for all four datasets. This experiment shows that using E-LargeVis to reduce the dimensionality of deep features not only reduces computational complexity and saves storage space, but also enhances the discriminative ability of deep features to obtain the optimal retrieval performance. 

## 7. Conclusions

In this paper, an extended LargeVis (E-LargeVis) dimensionality reduction method was proposed for high-resolution RSIR. Our E-LargeVis method uses SVR to fit the implicit mapping model of high-dimensional to low-dimensional by LargeVis, aiming at the deficiency of the original LargeVis method that is its inability to reduce the dimensionality of high-dimensional data of a single image. Next, a high-resolution RSIR method was proposed by using E-LargeVis to reduce the dimensionality of the fully connected layer extracted with SENet-ResNet50. We evaluated the proposed E-LargeVis dimensionality reduction method and other retrieval methods on four high-resolution remote sensing image datasets. The experimental results showed that the E-LargeVis method can greatly improve retrieval performance. In future work, we will try to use other fitting methods to further improve the performance of E-LargeVis. At the same time, it will be applied to other fields such as fine image classification to further verify its effectiveness. 

## Figures and Tables

**Table 1:** Deep feature dimensionality with different CNN networks.

	AlexNet	VGG-16	GoogLeNet	ResNet50	SENet-ResNet50
Dimensionality	4096	4096	1000	2048	2048

**Table 2:** Performance comparison of mean Average Precision (mAP) with different CNN backbone networks.

	AlexNet	VGG-16	GoogLeNet	ResNet50	SENet-ResNet50
UCM	77.19%	63.19%	74.97%	90.63%	96.64%
WHU-RS	72.00%	83.04%	70.43%	93.76%	97.69%
RSSCN7	81.60%	83.45%	83.57%	74.22%	85.10%
AID	69.57%	80.88%	63.51%	81.37%	89.03%

**Table 3:** Performance comparison of Support Vactor Regression (SVR) and other regression methods.

Method	UCM					WHU-RS				
	16	32	64	128	256	16	32	64	128	256
Ridge Regression	96.59%	96.45%	96.54%	96.39%	96.33%	97.19%	97.41%	97.20%	97.27%	97.33%
Lasso	95.68%	95.54%	95.63%	95.48%	95.42%	96.27%	96.49%	96.28%	96.35%	96.42%
SVR	98.27%	98.13%	98.22%	98.07%	98.01%	98.88%	99.11%	98.89%	98.96%	99.03%
**Method**	**RSSCN7**					**AID**				
	**16**	**32**	**64**	**128**	**256**	**16**	**32**	**64**	**128**	**256**
Ridge Regression	91.64%	91.92%	92.10%	92.03%	91.97%	92.95%	92.67%	92.93%	92.92%	92.74%
Lasso	90.79%	91.07%	91.24%	91.17%	91.11%	92.09%	91.81%	92.07%	92.06%	91.88%
SVR	92.22%	92.50%	92.68%	92.61%	92.55%	93.54%	93.26%	93.52%	93.51%	93.33%

**Table 4:** Performance comparison of Euclidean distance and other similarity measurement methods.

Dimensionality	Method	UCM	RS19	RSSCN7	AID
256	Euclidean	98.01%	99.03%	92.55%	93.33%
	Cityblock	96.15%	98.24%	93.13%	93.37%
	Chebychev	97.66%	98.25%	84.80%	92.82%
	Cosine	98.01%	99.03%	92.55%	93.33%
	Correlation	98.02%	99.02%	92.53%	93.34%
	Spearman	89.76%	95.04%	92.62%	92.42%
128	Euclidean	98.07%	98.96%	92.61%	93.51%
	Cityblock	97.66%	98.22%	90.99%	93.31%
	Chebychev	97.68%	98.74%	89.16%	93.25%
	Cosine	98.07%	98.96%	92.61%	93.51%
	Correlation	98.09%	98.95%	92.57%	93.51%
	Spearman	94.60%	96.75%	90.48%	93.02%
64	Euclidean	98.22%	98.89%	92.68%	93.52%
	Cityblock	98.07%	98.71%	91.42%	93.35%
	Chebychev	97.60%	98.30%	91.40%	93.24%
	Cosine	98.22%	98.89%	92.68%	93.52%
	Correlation	98.22%	98.92%	92.69%	93.52%
	Spearman	97.36%	96.86%	89.53%	93.03%
32	Euclidean	98.13%	99.11%	92.50%	93.26%
	Cityblock	98.22%	98.68%	91.85%	93.20%
	Chebychev	97.61%	98.62%	91.30%	93.04%
	Cosine	98.13%	99.11%	92.50%	93.26%
	Correlation	98.13%	99.13%	92.35%	93.25%
	Spearman	96.04%	95.15%	85.58%	93.37%
16	Euclidean	98.27%	98.88%	92.22%	93.54%
	Cityblock	98.26%	98.90%	92.02%	93.48%
	Chebychev	98.04%	98.23%	91.60%	93.42%
	Cosine	98.27%	98.88%	92.22%	93.54%
	Correlation	98.23%	98.74%	92.41%	93.51%
	Spearman	94.84%	66.44%	90.38%	84.06%

**Table 5:** Performance comparison of E-LargeVis and other dimensionality reduction methods.

Method	UCM					WHU-RS				
	16	32	64	128	256	16	32	64	128	256
PCA	97.29%	97.17%	96.91%	96.74%	96.70%	98.85%	98.39%	98.07%	98.01%	98.02%
LPP	96.94%	87.77%	73.50%	77.66%	86.53%	98.92%	89.59%	78.50%	83.72%	80.69%
LLE	67.73%	74.01%	81.04%	83.35%	85.85%	71.18%	83.80%	79.42%	82.47%	84.59%
E-LargeVis	98.27%	98.13%	98.22%	98.07%	98.01%	98.88%	99.11%	98.89%	98.96%	99.03%
**Method**	**RSSCN7**					**AID**				
	**16**	**32**	**64**	**128**	**256**	**16**	**32**	**64**	**128**	**256**
PCA	87.91%	87.01%	86.75%	86.70%	86.68%	88.56%	92.04%	90.54%	89.97%	89.71%
LPP	76.07%	76.45%	78.71%	85.54%	88.66%	82.44%	91.74%	82.64%	83.96%	85.21%
LLE	67.93%	77.41%	79.80%	73.51%	76.74%	56.00%	57.52%	64.49%	75.32%	79.35%
E-LargeVis	92.22%	92.50%	92.68%	92.61%	92.55%	93.54%	93.26%	93.52%	93.51%	93.33%

**Table 6:** Performance comparison with the existing methods.

Author	Year	UCM	WHU-RS	RSSCN7	AID
Hu, F. [16]	2016	62.23%	82.53%	-	-
Napoletano, P. [28]	2017	98.05%	98.69%	-	-
Zhou, W. [15]	2017	54.44%	64.60%	46.28%	37.61%
Xia, G. [17]	2018	64.56%	82.53%	59.04%	
Li, Y. [41]	2018	98.30%	-	-	-
Wang, Y. [25]	2019	90.56%	89.51%	81.32%	-
Ye, F. [31]	2019	95.62%	-	-	-
Liu, Y. [42]	2020	70.39%	-	-	-
Cao, R. [32]	2020	96.63%	-	-	-
Ours		98.27%	99.11%	92.68%	93.54%
